# IgG subclass switching and clonal expansion in cutaneous melanoma and normal skin

**DOI:** 10.1038/srep29736

**Published:** 2016-07-14

**Authors:** Louise Saul, Kristina M. Ilieva, Heather J. Bax, Panagiotis Karagiannis, Isabel Correa, Irene Rodriguez-Hernandez, Debra H. Josephs, Isabella Tosi, Isioma U. Egbuniwe, Sara Lombardi, Silvia Crescioli, Carl Hobbs, Federica Villanova, Anthony Cheung, Jenny L. C. Geh, Ciaran Healy, Mark Harries, Victoria Sanz-Moreno, David J. Fear, James F. Spicer, Katie E. Lacy, Frank O. Nestle, Sophia N. Karagiannis

**Affiliations:** 1 St. John’s Institute of Dermatology, Division of Genetics and Molecular Medicine, Faculty of Life Sciences and Medicine, King’s College London & NIHR Biomedical Research Centre at Guy’s and St. Thomas’s Hospitals and King’s College London, King’s College London, London SE1 9RT, United Kingdom; 2 Division of Cancer Studies, Faculty of Life Sciences and Medicine, King’s College London, 3rd Floor Bermondsey Wing, Guy’s Hospital, Great Maze Pond, London SE1 9RT, United Kingdom; 3 Breast Cancer Now Research Unit, Division of Cancer Studies, Faculty of Life Sciences and Medicine, King’s College London, 3rd Floor Bermondsey Wing, Guy’s Hospital, London, United Kingdom; 4 Tumour Plasticity Laboratory, Randall Division of Cell and Molecular Biophysics, New Hunt’s House, Guy’s Campus, King’s College London, London SE1 1UL, United Kingdom; 5 Skin Tumor Unit, St. John’s Institute of Dermatology, Guy’s Hospital, King’s College London and Guy’s and St Thomas’ NHS Trust, London, United Kingdom; 6 Wolfson Center for Age-Related Diseases; King’s College London, London, UK; 7 Department of Plastic Surgery at Guy’s, King’s, and St. Thomas’ Hospitals, London, United Kingdom; 8 Clinical Oncology, Guy’s and St. Thomas’ NHS Foundation Trust, London, United Kingdom; 9 Division of Asthma, Allergy and Lung Biology, Medical Research Council and Asthma UK Centre in Allergic Mechanisms of Asthma, Faculty of Life Sciences and Medicine, King’s College London, Guy’s Campus, London, United Kingdom

## Abstract

B cells participate in immune surveillance in human circulation and tissues, including tumors such as melanoma. By contrast, the role of humoral responses in cutaneous immunity is underappreciated. We report circulating skin-homing CD22+CLA+B cells in healthy volunteers and melanoma patients (n = 73) and CD22+ cells in melanoma and normal skin samples (n = 189). Normal and malignant skin featured mature IgG and CD22 mRNA, alongside mRNA for the transiently-expressed enzyme Activation-induced cytidine Deaminase (AID). Gene expression analyses of publically-available data (n = 234 GEO, n = 384 TCGA) confirmed heightened humoral responses (CD20, CD22, AID) in melanoma. Analyses of 51 melanoma-associated and 29 normal skin-derived IgG sequence repertoires revealed lower IgG1/IgG_total_ representation compared with antibodies from circulating B cells. Consistent with AID, comparable somatic hypermutation frequencies and class-switching indicated affinity-matured antibodies in normal and malignant skin. A melanoma-associated antibody subset featured shorter complementarity-determining (CDR3) regions relative to those from circulating B cells. Clonal amplification in melanoma-associated antibodies and homology modeling indicated differential potential antigen recognition profiles between normal skin and melanoma sequences, suggesting distinct antibody repertoires. Evidence for IgG-expressing B cells, class switching and antibody maturation in normal and malignant skin and clonally-expanded antibodies in melanoma, support the involvement of mature B cells in cutaneous immunity.

Despite being important immune sentinels in inflammation, antigen presentation, and adaptive immunity through antibody production, the recruitment and roles of B cells and the humoral immune compartment in cancer immune surveillance and in normal tissue homeostasis are insufficiently understood.

B cells that are exposed to antigens in peripheral tissues can undergo clonal expansion and class switching to mature antibody classes (IgG1-4, IgA1-2, IgE). Antigen challenge triggers daughter cells to undergo somatic hypermutation (SHM) and to express antibodies with increasing affinity for the specific antigen. Class switch recombination (CSR) and SHM, involving the enzyme Activation-induced cytidine Deaminase (AID) can occur both in lymph node germinal centers and also in tissues (e.g. lung, nasal mucosa) in response to antigenic challenge. This provides an enriched antibody repertoire with reactivity and affinity against encountered antigens and of different isotypes, conferring the potential to generate antibodies with a variety of Fc-mediated immune effector functions^1^,^2^,^3^.

The presence and nature of skin-resident B cells are ill-defined due to low cutaneous B cell infiltrate numbers. Preliminary findings describe a subset of B cells distinct from those in lymph nodes, circulating through sheep skin^4^. Moreover, potential roles for B cells in cutaneous inflammation, allergy and autoimmunity and in skin malignancy are reported^5^,^6^,^7^, suggesting immune surveillance in the context of inflammation or antigenic challenge in skin. In cutaneous melanomas, IgG-producing B cells may infiltrate tumors and form part of tertiary lymphoid structures^8^,^9^. Clonal expansion of IgG-expressing clones against tumor-associated antigens has been reported to correspond to clinical tumor regression^10^. Taken together, these studies support potential functions for mature humoral responses in normal and inflamed cutaneous sites.

We provide the first report of the human mature skin-resident B cell compartment and its IgG-expressing profiles in cutaneous melanoma and in normal skin. We describe evidence for the presence of cutaneous mature B cells, distinct IgG subclass distribution profiles, clonal expansion, somatic hypermutation in the IgG heavy chain variable regions, and predicted antigen binding site characteristics of the mature humoral response repertoire in cutaneous malignant melanoma lesions and in normal skin.

## Results

### B cells are present in melanoma lesions and normal skin

**Figure 1:**
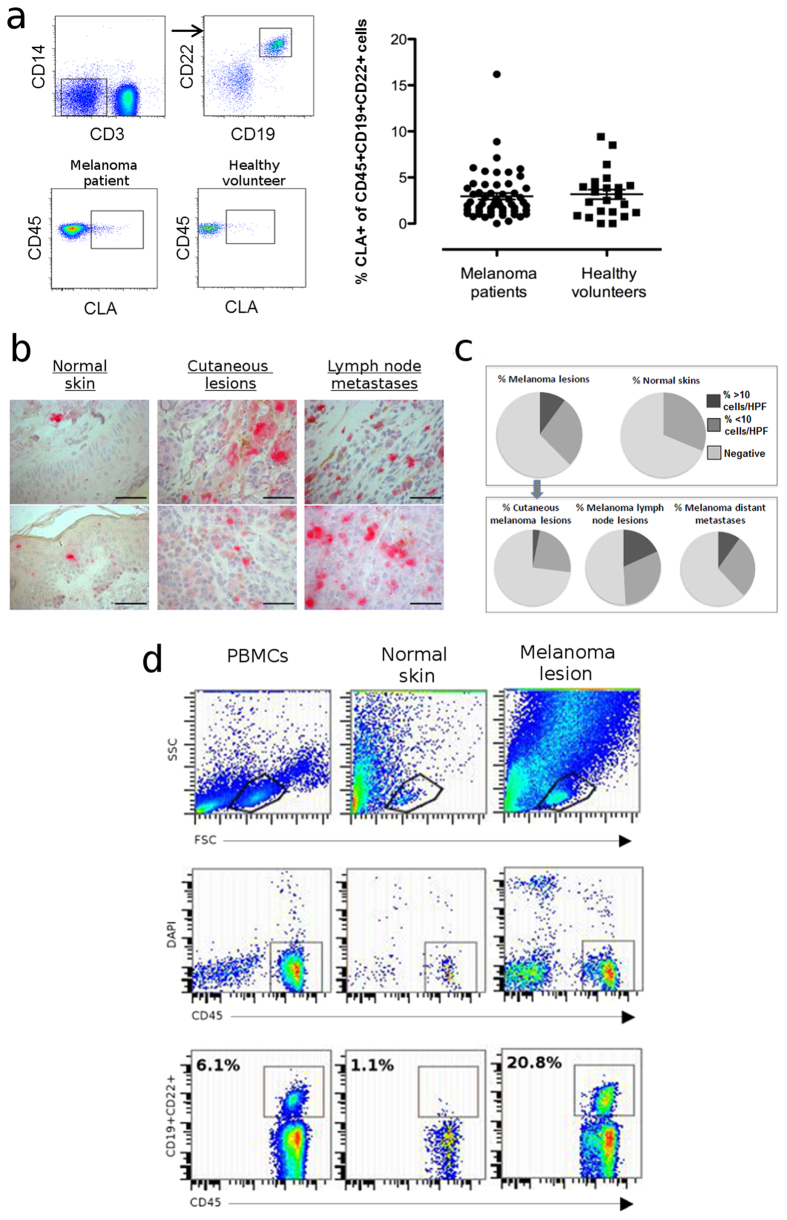
B cells may be recruited to skin and are present in melanoma lesions and normal skins. (**a**) Proportion (%) of circulating B cells positive for the skin homing marker CLA from patients with melanoma (n = 49) and healthy volunteers (n = 24), quantified by multi-color flow cytometric evaluations. Peripheral blood B cells were identified as CD3-CD14-CD19+CCD22+ cells (top panel). Representative dot plots of CLA+CD45+B cells from a melanoma patient and a healthy volunteer are shown (bottom panel). Quantification was based on % CLA+B cells from CD45+CD3-CD14-CD19+CCD22+PBMCs (right panel). (**b**) CD22+ cells in normal skin (left), cutaneous melanoma lesions (middle) and lymph node metastases (right) were detected by immunohistochemistry (tissue microarrays, top/bottom: example images per cohort, acquired on a Leica AxioScan, 40x objective; Scale bars: 66 μm). (**c**) CD22+ cell infiltrates per high-powered field (HPF) were quantified; Top panel: n = 189 melanomas, n = 16 normal skin samples; Bottom panel: CD22+ infiltrates stratified into cutaneous, lymph node and distant metastases. (**d**) B cells were detected from peripheral blood, melanoma lesion and normal skin samples using antibodies against CD19+, CD22+ (both FITC) and CD45+ (PE) and analysed by flow cytometry (example from matched blood, normal skin and cutaneous melanoma samples derived from a patient with melanoma).

We aimed to investigate B cell surveillance in cutaneous sites. We found that a small proportion of circulating CD45+CD3-CD14-CD19+CD22+B cells in healthy volunteers (n = 24) and patients with melanoma (n = 49) express the skin-homing Cutaneous Leucocyte-associated Antigen (CLA) (Fig. 1a, Figure S1a). Immunohistochemical evaluations revealed CD22+ cells in normal skins and melanomas (n = 189, Fig. 1b, Figure S1b). We detected low frequencies of CD22+ infiltrates in 31.3% of normal skin samples (n = 16). CD22+ infiltrates were found in 37.6% of melanomas (27% cutaneous lesions, 49.1% lymph node metastases, 38% distant metastases), with ~10% of melanomas featuring denser B cell infiltrating populations (>10 cells per high powered field, Fig. 1b,c). Cutaneous B cell infiltrates from non-malignant skin and melanoma lesion samples were also confirmed by flow cytometric analyses of CD45+CD19+CD22+B cells (Fig. 1d, for matched normal skin and melanoma lesion B cells and peripheral blood B cells from a single donor, representative of n = 4; Figure S2 for further examples of CD45+CD19+B cells from normal skin and melanoma lesion samples).

**Figure 2:**
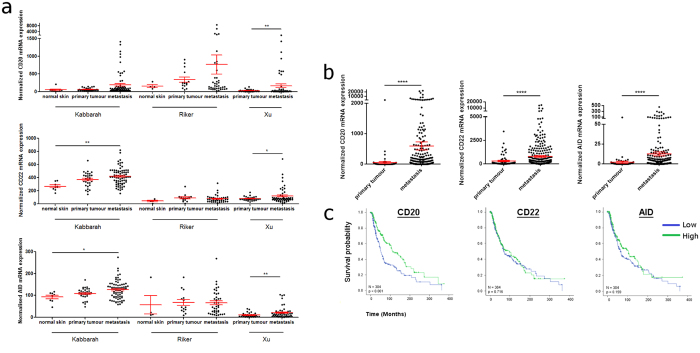
Upregulation of B cell markers in primary and metastatic melanomas compared with normal skins by gene expression analysis. (**a**) The relative expression of genes for the humoral immune markers CD20, CD22 and AID are elevated in primary and metastatic lesions compared with normal skin samples and also elevated in metastatic versus primary melanomas (n = 234, normalized microarray gene expression data from indicated studies, GEO database; One-way ANOVA with Tukey’s post hoc test). (**b**) Increased CD20, CD22, and AID relative expression in primary versus metastatic melanomas (n = 384, TCGA database; T-test with Welch’s correction was performed). (**a,b**) Graphs show mean ± SEM gene expression, *p < 0.05, **p < 0.01, ***p < 0.001, ****p < 0.0001. (**c**) Kaplan-Meier estimates of overall survival in TCGA melanoma patients according to CD20, CD22 and AID mRNA expression. Gene expression was categorized as low and high expression according to the median expression value and differences were compared using the log-rank test. High CD20 expression was significantly associated with better survival (n = 384; Hazard ratio (HR):1.82; 95% confidence interval (CI):1.36–2.43; p < 0.001). Detailed statistical analyses are described in Materials and Methods.

Analysis of publicly-available gene expression data extracted from human melanoma samples derived from Gene Expression Omnibus (GEO)^11^,^12^,^13^ further supports the expression of the genes for B cell markers MS4A1 (CD20) and CD22 and of the transiently-expressed SHM/CSR enzyme AICDA (AID) in normal skin, primary, and metastatic melanomas. All three B cell markers were upregulated in melanoma compared with normal skin samples and in metastatic compared with primary melanomas (n = 234, GEO database) (Fig. 2a). Increased relative expression of CD20, CD22 and AID in metastatic compared with primary melanoma were also found by analyses of the Cancer Genome Atlas (TCGA) database (Fig. 2b). Furthermore, a significant association between higher CD20 mRNA expression (p < 0.001) with longer patient survival (TCGA, n = 384, Fig. 2c) was observed, consistent with previous studies^14^,^15^,^16^.

Thus, B cells may be recruited to normal skin and cutaneous melanoma sites, based on expression of the humoral immune cell markers CD20, CD22 and AID.

### Biased IgG subclass distribution and antibody complementarity-determining region 3 (CDR3) lengths in cutaneous sites compared with IgG from circulating B cells

To determine the subclass distribution and heavy chain variable region profiles of antibodies expressed at cutaneous sites in our sample collection, we identified n = 51 unique mature IgG sequences from 9 cutaneous melanoma samples (patient characteristics in Table S1) and n = 29 unique mature IgG sequences from 13 normal skin samples.

**Figure 3:**
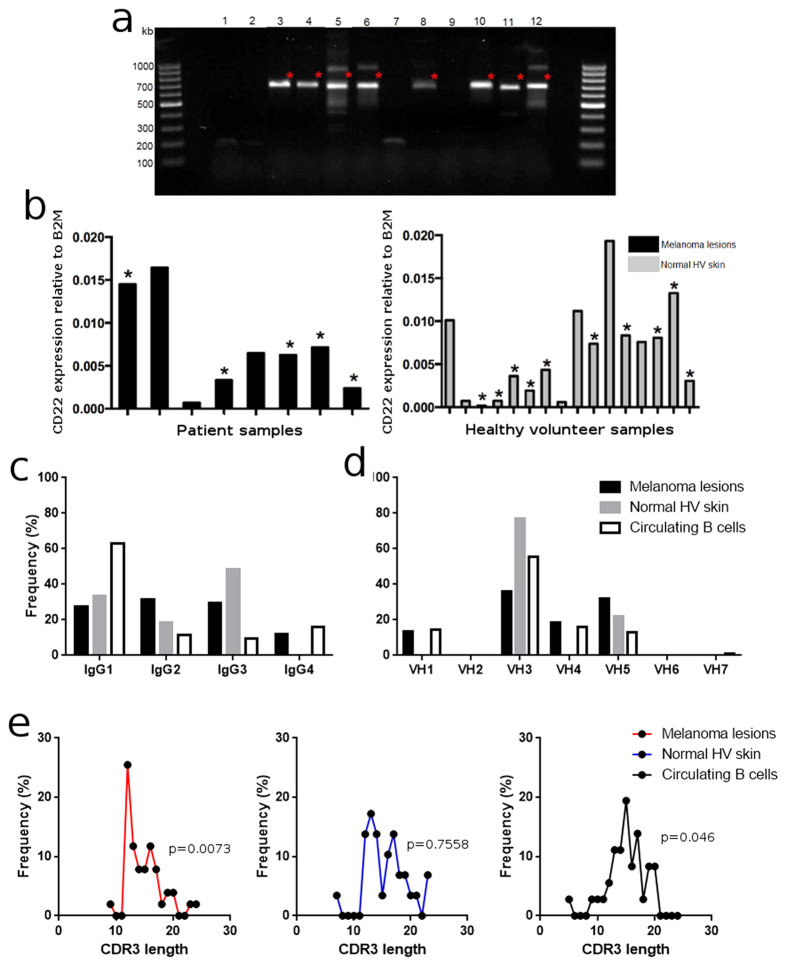
IgGVH, CD22, AID in human skin, IgG subclass and hypervariable region analyses support unique cutaneous IgG subclass and CDR3 length profiles. (**a**) IgGVH regions were amplified and mature IgG mRNA (red stars) detected. (**b**) In these samples, CD22 mRNA was detectable (qPCR) in melanoma lesion (black) and normal skin (grey) samples (black asterisks indicate samples expressing AID mRNA). (**c,d**) IgG subclass and VH family usage represented as the frequency from the total number of sequences (n = 51 cutaneous melanoma, n = 29 normal skin, n = 38 circulating B cell sequences, human BLAST) reveal specific cutaneous versus circulating IgG subclass profiles. (**e**) CDR3 hypervariable region analyses from melanoma lesions (n = 51, red), normal skins (n = 29, blue), melanoma patient circulating B cells (n = 38, black) (IMGT/V-quest database) suggest differential CDR3 length distribution in melanoma.

Samples with detectable IgG mRNA (Fig. 3a), expressed CD22 and some specimens from both groups also had detectable mRNA for the enzyme AID (Figs 3b and S3), suggesting the presence of a local cutaneous B cell and humoral response irrespective of malignant disease status.

We previously reported that B cells in melanoma feature distribution of IgG subclasses with lower representation of IgG1/IgG_total_ (40–45%) and proportionally-higher levels of IgG4/IgG_total_ (~20%) than those in human adult circulation at the protein level (65–80% IgG1, 2–4% IgG4)^8^,^17^,^18^. We examined 51 unique mature IgG sequences from 9 cutaneous melanoma samples, 29 unique mature IgG sequences from 13 normal skin samples and 36 unique mature IgG sequences randomly derived from circulating B cells of 12 patients with melanoma. From the molecular profiles of the mature IgG subclasses, 34% of the sequences detected from melanoma samples were IgG1, and 12% belonged to the IgG4 subclass (Fig. 3c). Normal skin samples displayed a similar distribution pattern, with lower representation of IgG1/IgG_total_ sequences (24%) (Fig. 3c, Table S2). These profiles were distinctly different to the IgG sequences (n = 36) from circulating B cells of patients with melanoma, in which IgG1 was the predominant subclass (62% IgG1). These findings indicate that B cell IgG subclass profiles in cutaneous sites feature a lower representation of IgG1 antibodies compared with those from B cells in the circulation.

In the same sample cohort (51 mature IgG sequences from cutaneous melanomas, 29 IgG sequences from normal skins and 36 IgG sequences from circulating B cells of patients), the sequences derived from cutaneous melanoma lesion B cells featured representation of immunoglobulin variable heavy chain region (IgVH) families (VH1-5), with highest representation from VH3 (36%) and VH5 (431) (Fig. 3d). IgG sequences from normal skins also showed only VH3 (78%) and VH5 (22%) family usage. The predominant families in circulating B cell sequences are VH3 (55%) and VH4 (16%). A closer inspection of the VH, VD and VJ gene segment usage showed a broad use of VH, VD, and VJ genes in patient circulating B cells. In sequences from B cells in melanoma lesions and healthy skin, we detected a preference for the usage of specific gene segments (red arrows, Figure S4), possibly suggesting a tissue-resident humoral compartment.

We then explored the lengths of the complementarity-determining CDR3 regions (IMGT/V-quest), the hypervariable areas largely responsible for determining antibody binding specificity and affinity to cognate antigens^19^. We found a double-peak distribution of CDR3 lengths in sequences from melanoma lesions (Fig. 3e), which suggests that these sequences are not sampled from a Gaussian distribution (p = 0.0073, D’Agostino-Pearson test) and that the melanoma-derived sequences appear to feature a group with shorter hypervariable regions than those found in blood.

This may suggest the rise of a specific group of B cell clones in malignant skin when compared to healthy volunteer skin and circulating B cells from melanoma patients^20^.

### IgVH sequences from melanoma and normal skins display comparable rates of mutation and clonal selection profiles

To assess the maturation state of the IgG antibodies expressed at cutaneous sites, we measured the silent and non-silent mutations which result in divergence from respective germline sequences.

**Figure 4:**
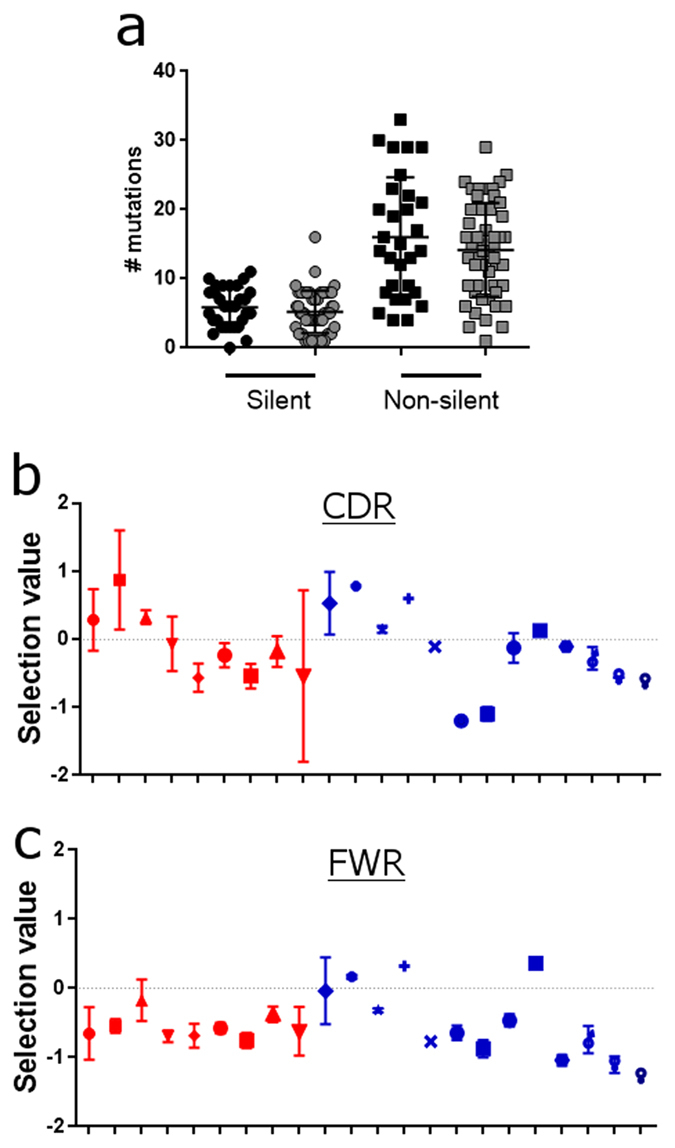
Similar mutational rates and clonal selection in melanoma and normal skin IgG antibodies. (**a**) Silent and non-silent mutations in the IgG VH regions determined using IMGT V-quest. Healthy volunteer skin (grey) and melanoma (black) samples show no significant differences in the antibody mutational rates. (b&c) Quantified clonal selection in the CDR (**b**) and FWR (**c**) regions from IgG VH sequences for each melanoma (red symbols ± SEM) and normal skin (blue symbols ± SEM) samples. NS: not significant. Please see Supplementary Materials and Methods (each symbol represents the mean value of selection from the sequences from each individual donor).

We found comparable mutagenic rates in melanoma and normal skin antibodies, suggesting similar levels of affinity maturation (Fig. 4a, Mann-Whitney test). Since affinity maturation and local clonal expansion may also be associated with negative clonal selection (editing)^21^, we quantified positive or negative selection of B cells by comparison of sequences to reference germline sequences using the BASELINe server. This determines the rate of mutation in specific areas of the VH sequence and therefore the survival and persistence of specific IgG VH sequence-expressing B cells *in situ*^22^. In the CDR regions (Fig. 4b), 63% of sequences from melanoma lesions and 63% from normal skin samples and in the framework regions (FWR) (Fig. 4c), 90% of sequences from melanomas and 76% from normal skin samples, were predicted to be undergoing negative selection (Figure S5).

Together, these suggest that the rates of mutation, and by extension, the maturation of the antibody variable region sequences, in normal skins and melanoma lesions appear comparable.

### Cutaneous melanoma- and normal skin-derived sequences display similar somatic hypermutation hotspot patterns, but distinct predicted binding sites

**Figure 5:**
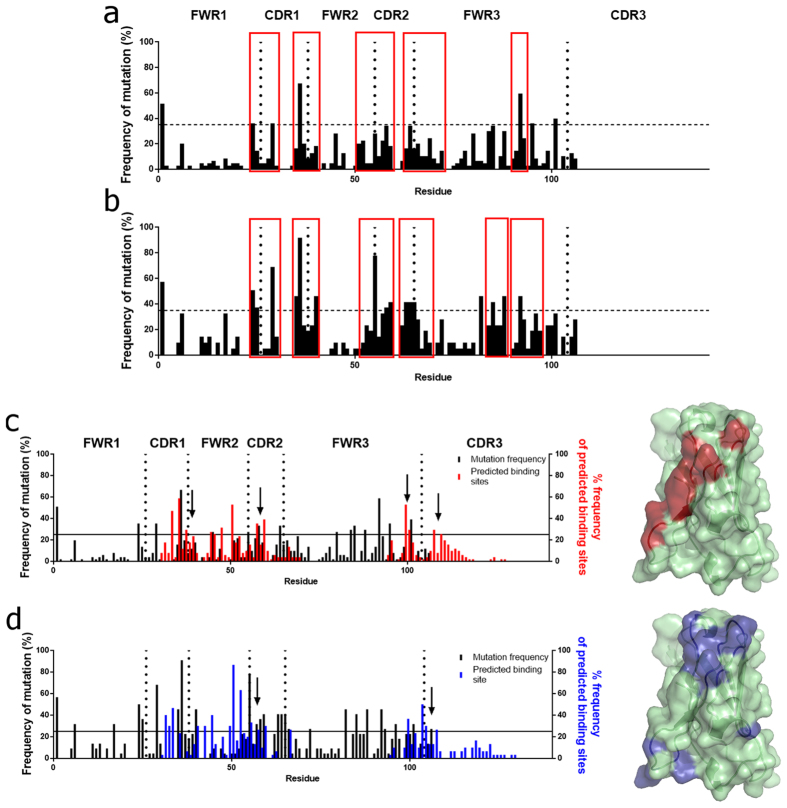
Somatic hypermutation frequency hotspots and predicted binding sites define distinct patterns in cutaneous melanomas and in normal skins. Divergence from germline sequences from melanomas (**a**), (n = 51) and normal skins (**b**) (n = 29) (red boxes: mutational clusters) (horizontal lines ≥ 35% identify somatic hypermutation ‘hotspots’). ((**c,d**), Left) Frequency of occurrence of predicted binding residues (n = 51 melanomas, red (**c**)), (n = 29 normal skins, blue (**d**), clusters indicated by arrows), superimposed on somatic hypermutation frequencies (black). Sequences compared to IMGT for non-silent mutations resulting in amino acid change; FWR and CDR: vertical dotted lines; horizontal lines at ≥25% identify predicted binding sites. ((**c,d**), Right) Residues predicted to participate in antigen recognition in ≥25% of sequences (red, melanomas; blue, normal skins) on a homology model of a V region sequence.

We examined the amino acid residue positions at which cumulative divergence from the germline occurred (incidence of non-silent mutation in >35% of sequences; threshold indicated by horizontal lines)^23^ (Fig. 5a,b). High densities of mutations from both cohorts cluster in areas bordering FWR1/CDR1, FWR2/CDR2, CDR2/FWR3 and in FR3 (red boxes). This signifies somatic hypermutation, supported by clonally-expanded sequences in melanoma and normal skin samples, featuring point mutation divergence from the germline (red) (Figure S6).

Furthermore, we examined individual residues predicted to be involved in antigen recognition (i-Tasser)^24^. We observed a higher concordance (overlap) between the rates of somatic hypermutation (black) and the predicted areas of antigen binding (red) in the melanoma-derived sequences, compared with overlap between somatic hypermutation frequency (black) and predicted binding residues (blue) in normal skin-derived sequences (Fig. 5c,d, left).

We then constructed homology models of the VH immunoglobulin fold, to highlight the residues predicted to likely be involved in antigen binding in >25% of the sequences from each cohort (above horizontal lines, Fig. 5c,d, left). These revealed different predicted areas of potential antigenic binding in melanoma-derived compared with normal skin-derived sequences (Fig. 5c,d, right). A single region of potential antigen binding was predicted in the 51 sequences from melanoma lesions, while several dispersed clusters of binding sites were predicted in normal skin sequences laid over the predicted variable region fold.

These data may indicate that different areas of the VH regions of tissue-resident B cell-derived antibodies from the two cohorts of melanoma and normal skin samples participate in F(ab) mediated binding.

### Evidence for antibody class switch recombination in melanomas and normal skin and clonal expansion in melanomas

**Figure 6:**
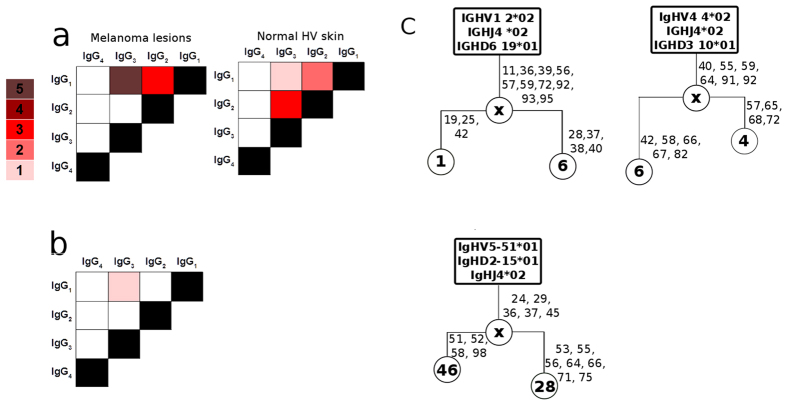
Cutaneous class-switched B cells in normal and melanoma skins and melanoma-associated clonally-expanded B cells. Number of V-D-J germline sequences displaying clonal expansion (IMGT V-quest) and switching to different IgG subclasses (human BLAST). (**a**) Class switching of identical V(D)J sequences from melanoma lesions (n = 6) and normal skins (n = 3) to different IgG subclasses. (**b**) Class switching of clonally-related amino acid sequences (n = 3), determined by identical V(D)J germline \‘mother\’ clones in the melanoma cohort only. (**c**) Clonal family trees constructed from melanoma lesion sequences display expansion from the same germline sequences. Germline indicated in rectangular boxes; circles with numbers indicate arbitrary number given to sequence; circles labeled ‘x’ represent hypothetical progenitor daughter cells; numbers next to clones indicate V-region amino acid positions where mutations are detected.

Expression of B cell markers, IgG subclasses and AID (Figs 1, 2, 3) at cutaneous sites, along with indications of negative clonal selection and variable region mutation hotpots (Figs 4 and 5) may signify local class switching to different IgG subclasses and B cell clonal expansion in skin. Consistent with this, in each cohort of melanoma and normal skin samples we found antibodies displaying identical V(D)J sequences that belonged to different subclasses (Fig. 6a). Taken together with the presence of AID, these findings may suggest *in situ* cutaneous class-switch recombination. Additionally, clonally-related sequences of separate IgG subclasses determined by identical V(D)J germline ‘parent’ clones were detected only in melanoma samples (three of nine samples) (Fig. 6b). B cell clonal family trees from 3 melanoma samples confirmed melanoma-resident clonal expansion and clonally-related sequences with point mutations at more than one residue (Fig. 6c); furthermore we also found in one of these families that there was antibody subclass switching alongside clonal expansion.

Together, these indicate active class switch recombination (CSR) to different IgG subclasses in normal and malignant cutaneous sites, alongside B cell clonal expansion in melanoma lesions only, pointing to humoral responses against local immune and inflammatory signals or antigenic challenge.

## Discussion

We present evidence supporting the presence of mature, skin-resident IgG antibody-expressing B cells. Our findings add to previous reports of IgG-expressing B cells in melanoma lesions, B cells from patients that express antibodies reactive to tumor cells, and dysregulated levels of IgG subclasses in melanoma lesions and patient sera^14^,^16^.

Despite previously-reported positive associations between tumor-associated B cells and better clinical prognosis in melanoma^15^,^25^,^26^,^27^, here supported by our TCGA database gene expression analyses, the recruitment and roles of B cells and especially of the mature cutaneous humoral immunity remain insufficiently uncharacterized. Therefore, in our study we focused on analyzing B cells, the transiently-expressed class switch recombination and somatic hypermutation enzyme AID and mature IgG antibody sequences from cutaneous B cells in order to evaluate maturation and isotypes of locally-expressed antibodies.

Subsets of circulating (CD22+) B cells in patients with melanoma and healthy volunteers harbor skin homing potential, based on expression of CLA^28^. In concordance, we detected cutaneous B cells by flow cytometry, immunohistochemistry and qPCR. Mature IgG mRNA in skin and skin tumor lesions and mRNA for the transiently-expressed SHM/CSR enzyme, AID, point to a mature activated cutaneous humoral immune compartment with respect to antibody affinity maturation and class switching potential.

Humoral immunity can mount antibody responses through recognition of upregulated cancer-associated antigens, but conversely, cross-talk with tumors may educate these responses to adopt regulatory functions^29^. This may be manifested by tumor states supporting expression of regulatory antibody subclasses such as IgG4, normally associated with inflammation or immune regulation^30^,^31^,^32^. In line with this and with our previous findings at the protein level, we confirmed that melanoma-associated IgG subclass expression distribution lacks a predominant IgG1 representation and instead features proportionally-higher regulatory subclass IgG4 sequences (~20% IgG4/IgG_total_). Surprisingly, we detected a lower than expected proportional IgG1/IgG_total_ expression profile in normal skin samples. Dysregulated levels of IgG4 were also expressed by patient blood B cells, consistent with previous reported findings^33^. These are in contrast to well-documented IgG subclass representation in normal adult circulating and lymph node-resident B cells, where IgG1 is the predominant subclass (65–80% IgG1, 2–4% IgG4)^17^,^18^.

The presence of a skin-resident IgG-expressing cutaneous humoral compartment is not entirely unexpected. B cells are reported in autoimmune conditions with cutaneous pathogenic manifestations such as Pemphigus vulgaris, where B cells and auto-reactive antibodies are detected in skin and systemically, contributing to disease pathology^34^. IgG4 is implicated in autoimmune and inflammatory cutaneous diseases, including Pemphigus vulgaris, where deposits of IgG4 (and IgE) auto-antibodies home in to skin lesions. IgG2, which we also detected in our skin cohorts, is normally associated with responses to bacterial antigens, to which the skin may be commonly exposed^35^,^36^,^37^,^38^,^39^,^40^. These skin-resident IgG subclass profiles without a predominant IgG1 representation may therefore be consistent with a role for B cells in maintaining local homeostasis, and responding to local pathogen challenge^41^,^42^.

We observed an active B cell compartment in melanoma lesions, supported by different findings and these, along with gene expression analyses of published data, implicate tissue-resident B cells and AID expression in primary and in metastatic melanomas. A subset of melanoma-resident and perhaps skin-resident B cell clones displayed different CDR3 lengths when compared with those from the circulation, indicating humoral immune responses focused towards a specific antigenic stimulus. Consistent with antigen-focused clonal expansion, homology modeling of melanoma-derived antibodies suggested structural clustering of amino acid residues highly predicted to be involved in binding. This may indicate antibody maturation under clonal selection pressure in melanoma. Furthermore, we found active class switching in identical B cells in normal and in malignant cutaneous sites. Importantly, we found class switching in clonally-related B cells and clonal family trees in melanomas but not in normal skin samples, possibly associated with responses to tumor antigens and tumor evolution^43^,^44^. Therefore, the combination of affinity maturation, class switch recombination and clonal expansion might be a feature of melanoma-associated antibodies, consistent with active and heightened humoral responses to a specific antigen repertoire^45^.

In summary, we report basal levels of a mature humoral immune response in normal skins and an enhanced B cell clonal presence in cutaneous melanomas. Skin tumor-associated B cell receptor repertoires and IgG subclass profiles point to an active tumor-infiltrating B cell compartment with features distinct to those of circulating B cells. Our findings of local B cell clonal expansion, antibody affinity maturation and predicted antigenic binding patterns on antibody variable regions also raise the possibility that a heightened mature IgG antibody response in melanoma may be edited towards specific antigen stimuli. Melanoma may activate but also pervert skin-resident humoral immune response homeostasis to control activated, mature B cell responses. This may be through maintaining skin-resident antibody subclass distribution featuring immune regulatory subclass antibodies such as IgG4, as a mechanism of evading immune surveillance.

Our data thus report a humoral response in skin immune surveillance and homeostasis and support expanded tumor-associated humoral immunity in cutaneous melanoma. These findings mandate a closer study of the cutaneous humoral immune cell compartments to elucidate the involvement of B cells and locally-expressed antibodies in skin immune surveillance and their roles in melanoma tumor pathology.

## Materials and Methods

### Sample collection and characterization of human B cells

Patients with malignant melanoma were staged and classified according to the American Joint Committee on Cancer Melanoma Staging and Classification criteria^46^. Discarded human skin samples were obtained from volunteers undergoing routine plastic surgery. Specimens were collected with informed consent in accordance with the Helsinki Declaration (approved by the Guy’s Research Ethics Committee, Guy’s and St. Thomas’ NHS Foundation Trust). Samples were used freshly or placed in RNAlater (Life Technologies) and stored at −70 °C.

For evaluations of CLA+B cells, peripheral blood mononuclear cells (PBMC) were isolated by Ficoll-Paque Plus density centrifugation GE Healthcare). Cells were resuspended in RPMI 1640 medium (Life Technologies) 11.25% human serum albumin (Gemini Bio-Products, West Sacramento, CA), 10% DMSO (Sigma) and stored in liquid nitrogen. For analyses, cells were thawed, selected based on SSC-A/FSC-A properties, dead cells excluded using LIVE/DEAD® Fixable Dead Cell Stains in Qdot585 (Life Technologies) and single cells selected by FSC-A/FCS-H. Cells were incubated with antibodies for the following surface markers (BD Biosciences): CD45(V500), CD22 (APC), CD19 (BV605), CD3 (APC-Cy7), CD14 (Alexa Fluor 700) and CLA (FITC). Samples were subsequently acquired on a 5-laser SORP Fortessa (BD Bioscience) and data analyzed using FlowJo software (Treestar).

Single cell suspensions from normal skin and cutaneous melanoma samples were obtained by mild physical dissociation (Gentle MACS, Miltenyi Biotec). Cells were allowed to crawl out from tissues overnight in RPMI 1640 medium, 10% FCS, at 37 °C, 5% CO_2_. Tissues were filtered through a 100 μm strainer. B lymphocytes were also identified from matched peripheral blood samples after Ficoll-Paque (GE Healthcare) gradient centrifugation. Cells were treated with FcR Blocking Reagent (Miltenyi Biotec), selected based on SSC-A/FSC-A properties, DAPI to exclude dead cells and labeled with anti-human antibodies to the following markers: CD19 (FITC), CD22 (FITC), CD45 (PE) or with CD19 (FITC) and CD45 (PerCP) (BD Biosciences) to detect B cells by flow cytometry (FACSCanto, BD Biosciences). Data were analyzed using FlowJo software (Treestar).

### Immunohistochemical evaluations of CD22+ cells

To detect CD22 expression, 6–8 μM-thick melanoma tissue microarray (ME2802, US Biomax) samples were de-paraffinized in xylene before rehydration. Heat antigen retrieval and staining with mouse anti-human CD22 (Abcam) were followed by biotinylated goat/rabbit anti-mouse IgG (DAKO) and detection using Vector red alkaline phosphatase substrate with levamisole (Vector labs). Images were acquired using ZeissAxioScan microscope with 40x objective and Olympus Provis stereology microscope with 40x planapo objective, and analyzed using the Zen Blue software (Leica).

### RNA extraction and cDNA synthesis to identify IgG-expressing cutaneous specimens

Ti**s**sues stored in RNAlater were thawed at room temperature, homogenized in RLT buffer (RNeasy kit, QIAgen) using a Kinematica Polytron homogenizer (Fischer Scientific). RNA was extracted using the RNeasy kit (QIAgen) and quantified using a Nanodrop spectrophotometer (Thermo Scientific). 500 ng RNA was used to synthesize cDNA using SuperScript ® II reverse transcriptase (Life Technologies). RNA extracted from 27 normal skin and 21 melanoma samples yielded n = 29 IgG sequences from 13 normal skins and n = 51 IgG sequences from 9 melanoma samples; RNA from n = 36 single B cell IgG sequences was obtained from PBMC samples of 12 patients with melanoma using a single cell lysis kit (Ambion, ThermoFisher) following manufacturer recommendations. cDNA synthesis was conducted using the SuperScript VILO cDNA Synthesis kit (Invitrogen, ThermoFisher).

### Quantitative PCR analysis of cell marker expression

cDNA was synthesized and used in conjunction with a FAM-labelled Taqman gene expression kit for human CD22 (Hs00998488) and AID (Hs00757808), with VIC-labeled β2-microglobulin as endogenous control (Life Technologies); 384-well plates were used with the 7900HT system (Applied Biosystems) to amplify CD22 and AID. Endogenous control and mRNA abundance were normalized against human β2-microglobulin mRNA (VIC) (2^ΔCt^). AID expression was normalized against CD22 mRNA (VIC) (2^ΔCt^).

### Semi-Nested PCR to amplify VH-Cγ transcripts and sequencing VH regions

Amplification of VH-Cγ transcripts consisted of 2 rounds of semi-nested PCR: 1st reaction used 2 μL of the cDNA and 6 leader sequence PCR forward primers (VH_1_L-VH_6_L), along with an IgGPr reverse primer at 0.5 μM in a PCR reaction with high fidelity polymerase master mix Phusion Flash (Thermo Scientific) (Table S3); 2^nd^ reaction proceeded with Phusion Flash mix using 2 μL of the first reaction with class-specific PCR forward primers (VH_1_F-VH_6_F) and the same IgGPr reverse primer as in 1st reaction (at 0.5 μM). VH-Cγ transcripts were identified by (1%) agarose gel electrophoresis (650 bp). Fragments were isolated by band excision and DNA extraction (QIAgen gel extraction kit, QIAgen). DNA was cloned by blunt-ended ligation into pCR-Blunt from the Zero Blunt® cloning kit (Life Technologies). Ligation reactions were transformed into chemically-competent *E. coli* OneShot™ TOP10 cells (Life Technologies). Colonies were selected and treated for pCR-Blunt plasmid extraction (QIAgen plasmid miniprep kit). Plasmids were sequenced using the M13 forward and reverse sequencing primers (Source Bioscience) (Table S4).

### IgG sequence analyses

Cloned IgG V-region sequences were obtained from sequence reactions using the FinchTV software (PerkinElmer). Forward and reverse IgG sequences were analyzed using IMGT/V-Quest to determine IgG heavy chain family, V-D-J germline, and hypermutation sites^47^. Selectivity of the cloned V-regions was determined using the BASELINe server according to the IMGT numbering strategy^22^, http://selection.med.yale.edu/baseline/).The Baseline server compares expected versus observed rates of mutation to quantify selective pressure; a binomial test is used to quantify statistical significance. IgG subclass was determined by comparing the reverse IgG variable region sequence in the NCBI database using human BLAST^48^; alignments displaying highest identity with greatest coverage and ≥90% sequence match to a specific subclass and following confirmation with described subclass amino acid patterns were included^49^. Amino acid sequences of isolated V-D-J regions were summed across all sequences for each cohort and plotted according to residue number and submitted in entirety to the i-Tasser database to evaluate structure and areas of predicted binding 24.

### Analysis of B cell marker gene expression from human databases

Publicly-available data from n = 234 human skin and melanoma samples were extracted from the Gene Expression Omnibus (GEO) database (GSE7553, n = 58)^12^; (GSE46517, n = 93)^11^; (GSE8401, n = 83)^13^. Data were normalized using Gene Pattern software^50^.

Gene expression data of n = 384 human melanoma samples from The Cancer Genome Atlas (TCGA) database (http://cancergenome.nih.gov/) were also used to analyze CD20, CD22 and AID mRNA expression in melanoma progression. We only considered samples from patients who had not received neo-adjuvant treatment prior to tumor resection for the sample submitted for TCGA. Normalized mRNA expression data were downloaded from cBioPortal^51^. Gene expression data analyses were presented as mean ± standard error of the mean (SEM). Statistical analyses are described below.

### Statistical analyses

GraphPad Prism software (GraphPad Software, www.graphpad.com) was used for statistical analyses (One-way ANOVA with Tukey’s post hoc test and T-test with Welch’s correction for determination of relative gene expression of GEO database and of TCGA database respectively; Mann-Whitney test for B cell distribution and analysis of associated markers with cancer progression; ANOVA to determine differences in mutational rates; Kolmogorov-Smirnov goodness-of-fit; D’Agostino & Pearson normality test to analyze CDR3 length distribution). For survival analyses, mRNA expression data from TCGA samples were categorized as low expression and high expression using the median expression as cut-off. Kaplan Meier survival curves were compared among patient subsets using the log-rank test. The Hazard Ratio (HR) was calculated using the Cox proportional hazard model. Survival analyses were performed using the SPSS software (IBM; http://www-01.ibm.com/software/uk/analytics/spss/). Differences with P-values < 0.05 were considered statistically significant and all tests were two-sided (*p < 0.05, **p < 0.01, ***p < 0.001, ****p < 0.0001).

## Additional Information

**How to cite this article**: Saul, L. *et al*. IgG subclass switching and clonal expansion in cutaneous melanoma and normal skin. *Sci. Rep.* **6**, 29736; doi: 10.1038/srep29736 (2016).

## Supplementary Material

Supplementary Information
