# Supplementary material for: IgG subclass switching and clonal expansion in cutaneous melanoma and normal skin
Source: Sci Rep. 2016 Jul 14;6:29736. doi: 10.1038/srep29736 (PMC4944184; doi:10.1038/srep29736)
Supplement: Supplementary Information [file srep29736-s1.doc]

**Supplementary information**

**IgG subclass switching and clonal expansion in cutaneous melanoma and normal skin**

Louise Saul^1,2*^, Kristina M. Ilieva^1,3*^, Heather J. Bax^1,2^, Panagiotis Karagiannis^1^, Isabel Correa^1^, Irene Rodriguez-Hernandez^4^, Debra H. Josephs^1,2^, Isabella Tosi^1^, Isioma U. Egbuniwe^1^, Sara Lombardi^1,5^, Silvia Crescioli^1^, Carl Hobbs^6^, Federica Villanova^1^, Anthony Cheung^1,3^, Jenny L.C. Geh^7^, Ciaran Healy^7^, Mark Harries^8^, Victoria Sanz-Moreno^4^, David J. Fear^9^, James F. Spicer^2^, Katie E. Lacy^1,5^, Frank O. Nestle^1^, Sophia N. Karagiannis^1^

^1^ St. John’s Institute of Dermatology, Division of Genetics and Molecular Medicine, Faculty of Life Sciences and Medicine, King’s College London & NIHR Biomedical Research Centre at Guy's and St. Thomas's Hospitals and King's College London, King’s College London, London SE1 9RT, United Kingdom

^2^ Division of Cancer Studies, Faculty of Life Sciences and Medicine, King’s College London, 3rd Floor Bermondsey Wing, Guy’s Hospital, Great Maze Pond, London SE1 9RT, United Kingdom

^3^ Breast Cancer Now Research Unit, Research Oncology, Faculty of Life Sciences and Medicine, King’s College London, 3rd Floor Bermondsey Wing, Guy's Hospital, London, United Kingdom

^4^Tumour Plasticity Laboratory, Randall Division of Cell and Molecular Biophysics, New

Hunt’s House, Guy’s Campus, King’s College London, London SE11UL, United Kingdom

^5^ Skin Tumor Unit, St. John’s Institute of Dermatology, Guy’s Hospital, King’s College London and Guy's and St Thomas' NHS Trust, London, United Kingdom

^6^ Wolfson Center for Age-Related Diseases; King’s College London; London, UK

^7^ Department of Plastic Surgery at Guy’s, King’s, and St. Thomas’ Hospitals, London, United Kingdom

^8^ Clinical Oncology, Guy’s and St. Thomas’ NHS Foundation Trust, London, United Kingdom

^9^ Division of Asthma, Allergy and Lung Biology, Medical Research Council and Asthma UK Centre in Allergic Mechanisms of Asthma, Faculty of Life Sciences and Medicine, King’s College London, Guy’s Campus, London, United Kingdom

* Equal contribution

Correspondence:

Sophia N. Karagiannis, PhD, St. John’s Institute of Dermatology, Division of Genetics and Molecular Medicine, Faculty of Life Sciences and Medicine, King’s College London& NIHR Biomedical Research Centre at Guy’s and St. Thomas’s Hospitals and King’s College London, Guy’s Hospital, Tower Wing, 9th Floor, London, SE1 9RT, United Kingdom

E-mail: [sophia.karagiannis@kcl.ac.uk](mailto:sophia.karagiannis@kcl.ac.uk)

**
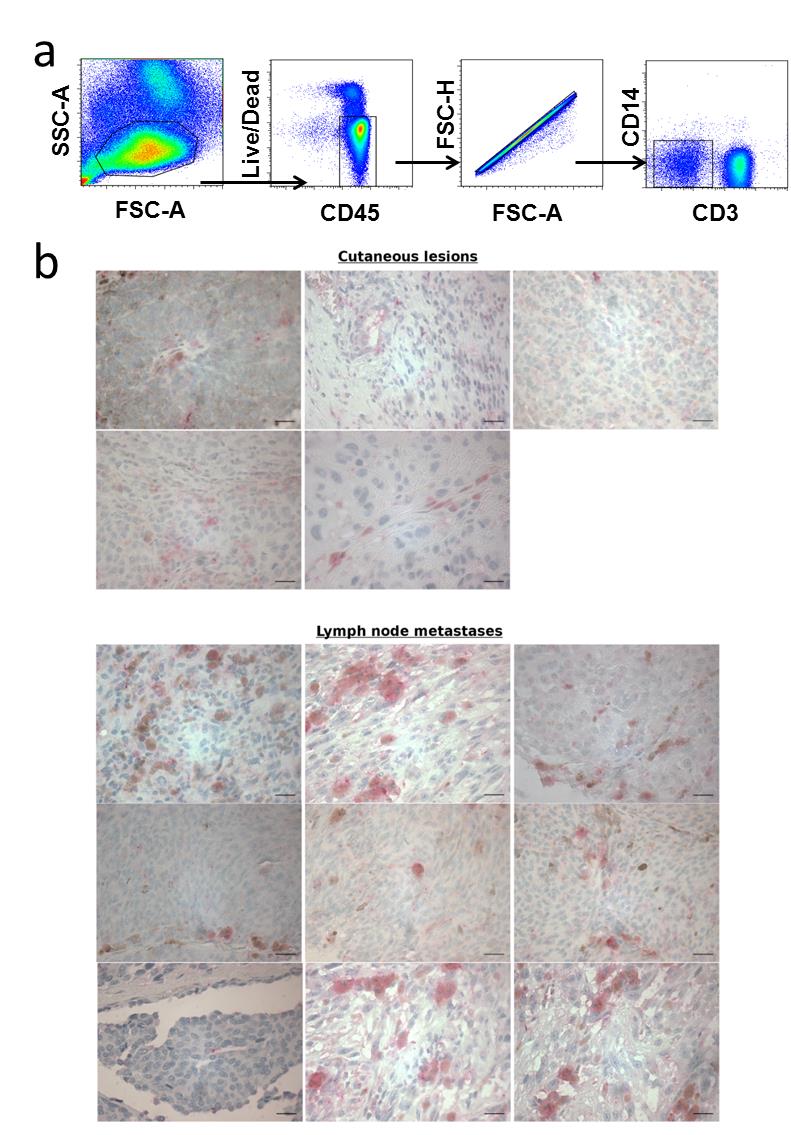
**

**Supplementary Figure S1:** a: Gating strategy to detecting circulating CLA+ B cells in human blood. Peripheral blood mononuclear cells (PBMC) were identified based on SSC-A/FSC-A properties, dead cells were excluded using LIVE/DEAD® Fixable Dead Cell Stains and single cells were selected by FSC-A/FCS-H. CD3-negative and CD14-negative cells were then gated and CD45+CD19+CD22+ B cells were studied to identify CLA+ cells (see Figure 1a). b: Examples of B cells in melanoma tumor lesions. Immunohistochemical evaluations of CD22+ cells in cutaneous melanoma lesions (top panels) and lymph node metastases (bottom panels) in tissue microarray specimens (acquired on a Leica AxioScan, with a x40x objective; Scale bars: 30 µm).


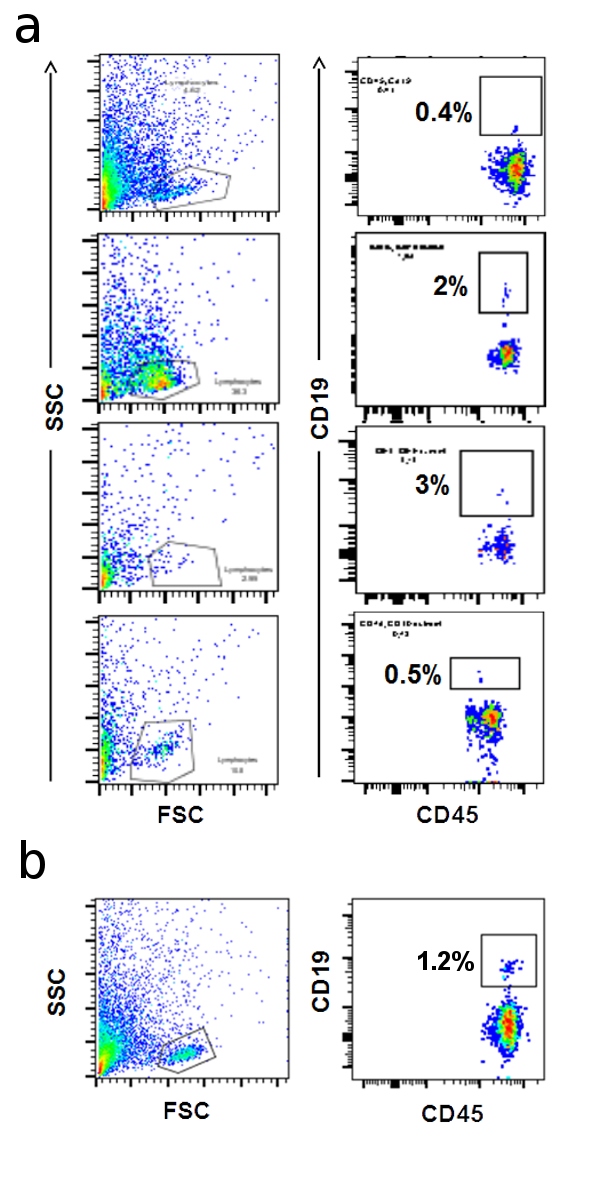


**Supplementary Figure S2:** Examples of CD19+CD45+ (CD19-FITC and CD45-PerCP) B cell populations (right) derived from lymphocyte infiltrates (gated, left) from normal skin samples **(a)** and from a cutaneous melanoma sample **(b)** detected by flow cytometry.

**
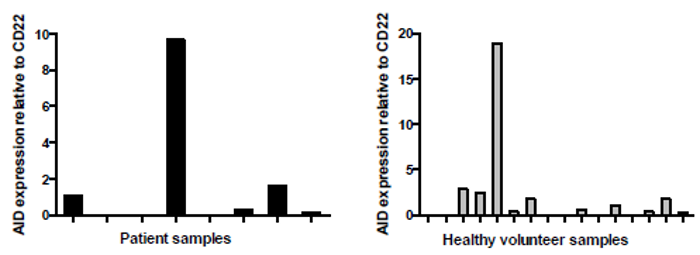
**

**Supplementary Figure S3:** mRNA for the transiently-expressed enzyme Activation-induced cytidine deaminase (AID) was detected in 5/8 and 10/16 melanoma lesions (black, left) and normal skin samples (grey, right), respectively, by quantitative PCR. Expression was normalized against CD22 mRNA.

**
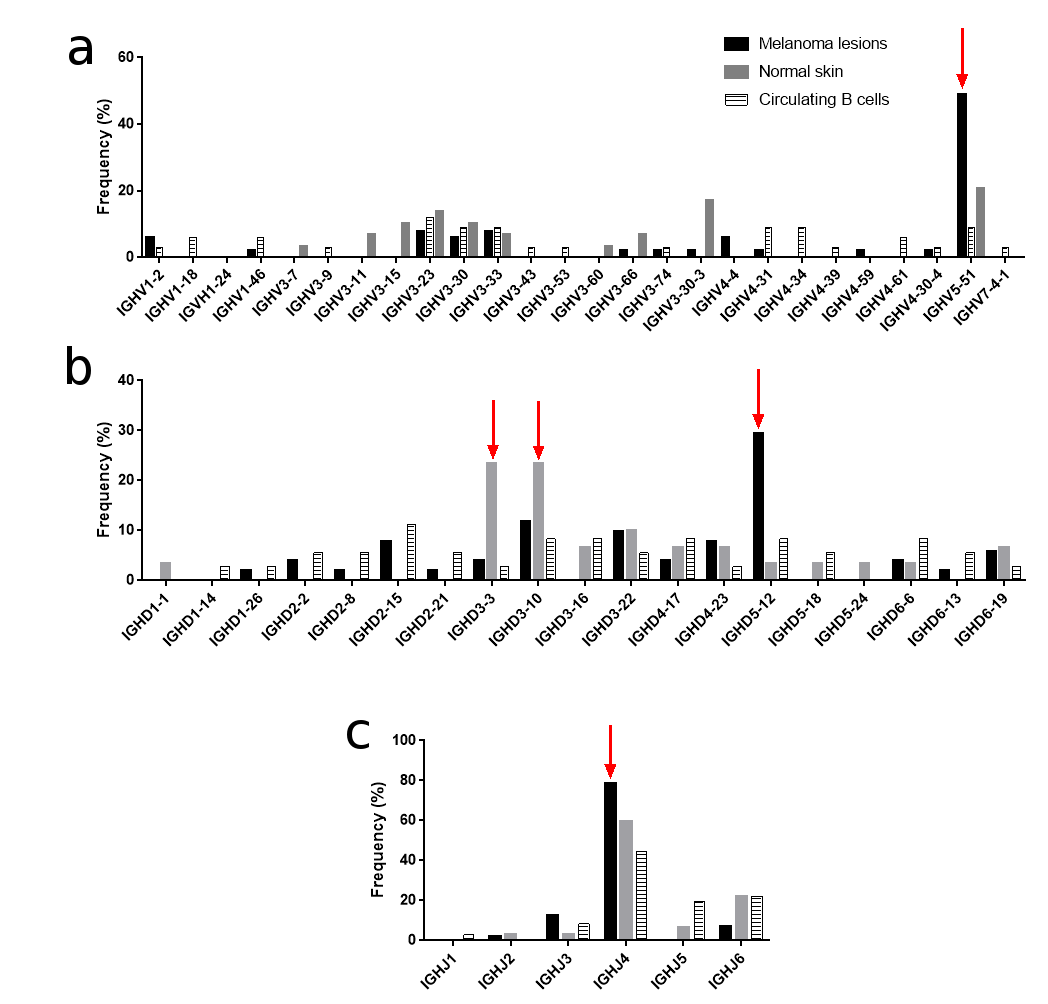
**

**Supplementary Figure S4:** IgG heavy chain usage in melanoma lesions, healthy skin and patient blood B cells. Patterns of IgG heavy chain VDJ family usage by comparison of variable regions of IgG heavy chains with sequences in the IMGT/V-quest database: IGHV (a), IGHD (b) and IGHJ (c) germline usage of sequences from melanoma lesions (black), normal skin samples (grey) and circulating B cells from patients with melanoma (striped) (n=51 sequences isolated from melanoma lesions, n=29 from normal skin of healthy volunteers, and n=36 sequences identified from circulating B cells of melanoma patients). Red arrows indicate gene segments which appear to be more frequently represented in sequences from B cells from melanoma lesions and healthy skins compared to those from circulating B cells.


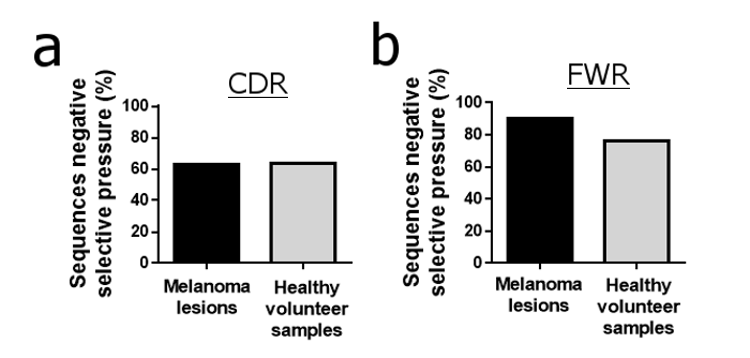


**Supplementary Figure S5:** Clonal selection of antibodies derived from melanoma lesions and normal skin samples. The proportion of pooled sequences displaying negative selection (from the germline) which were derived from melanoma were 63% of CDR (a, black) and 90% of FWR (b, black) sequences; in normal skin sequences: 63% of CDR (a, grey) and 76% of FWR (b, grey) sequences displayed negative selection.


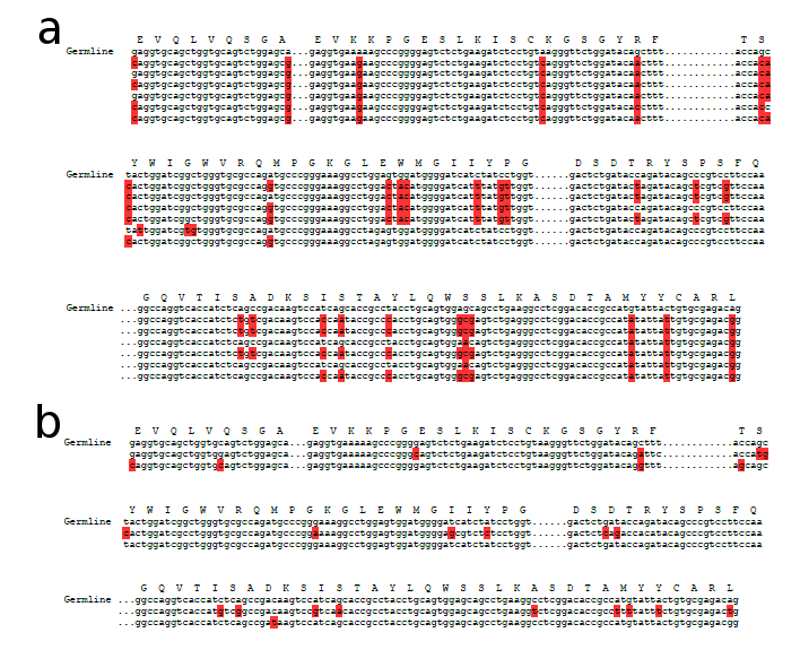


**Supplementary Figure S6:** Sequence alignments of clonally-expanded VHDJ region sequences indicating divergence from the germline. Examples of clonally-expanded VHDJ region sequences amplified by RT PCR derived from a cutaneous melanoma lesion sample (a) and from a normal skin specimen (b). Sequence divergence is indicated by point mutations (DNA substitutions are highlighted in red) from the germline (germline VH sequence IGHV5-51*01 extracted from the IMGT V-Quest database).

**Supplementary Table S1.** Clinical characteristics of melanoma patient study participants with IgG-positive cutaneous tumors.

|  | **Diagnosis** | | | **Sample information** | | | |
| --- | --- | --- | --- | --- | --- | --- | --- |
| **Patient** | Date of Diagnosis | Staging at Diagnosis | Age | Lesion type | Staging at biopsy | Breslow thickness (mm) | Presence of Ulceration |
| M1 | 2003 | IIIA | 78 | Metastases | IV | 3.12 | Absent |
| M2 | 2009 | IIA | 70 | Primary | IIB | 2.85 | Absent |
| M3 | 2009 | IB | 82 | Primary | IB | 1.08 | Absent |
| M4 | 1999 | IIIA | 48 | Metastases | IIIC | 1.12 | Absent |
| M5 | 1998 | IIB | 87 | Metastases | IV | 2.2 | Present |
| M6 | 2010 | IIC | 87 | Metastases | IV | 9 | Present |
| M7 | 2010 | IIIA | 59 | Primary | IIIA | 4.62 | Absent |
| M8 | 2007 | IIC | 75 | Metastases | IIIC | 6.3 | Present |
| M9 | 2012 | IIB | 76 | Primary | IIB | 2.4 | Present |

**Supplementary Table S2.** Sequences obtained from samples from the cohort of melanoma patients (M; top) and from the skin of healthy volunteers (HV; bottom).

| **Patient/ healthy volunteer ID** | **Total numbers of sequences** | **Numbers of sequences belonging to clonal families** | **Numbers of IgG1 sequences** | **Numbers of IgG2 sequences** | **Numbers of IgG3 sequences** | **Numbers of IgG4 sequences** | **Sequences not assigned an IgG subclass** |
| --- | --- | --- | --- | --- | --- | --- | --- |
| **M1** | 3 | 2 | 0 | 3 | 0 | 0 |  |
| **M2** | 4 | 3 | 3 | 0 | 1 | 0 |  |
| **M3** | 21 | 20 | 10 | 2 | 7 | 0 |  |
| **M4** | 4 | 0 | 2 | 0 | 2 | 0 |  |
| **M5** | 9 | 3 | 6 | 0 | 3 | 0 |  |
| **M6** | 7 | 0 | 2 | 1 | 2 | 0 | 1 |
| **M7** | 3 | 0 | 0 | 2 | 1 | 0 |  |
| **M8** | 4 | 0 | 0 | 1 | 0 | 2 | 1 |
| **M9** | 2 | 0 | 2 | 0 | 0 | 0 |  |
| **HV1** | 2 | 2 | 1 | 1 | 0 | 0 |  |
| **HV2** | 2 | 0 | 1 | 0 | 0 | 0 | 1 |
| **HV3** | 3 | 0 | 0 | 1 | 2 | 0 |  |
| **HV4** | 4 | 2 | 0 | 1 | 3 | 0 |  |
| **HV5** | 1 | 0 | 0 | 1 | 0 | 0 |  |
| **HV6** | 2 | 2 | 0 | 0 | 2 | 0 |  |
| **HV7** | 3 | 0 | 1 | 1 | 1 | 0 |  |
| **HV8** | 1 | 0 | 0 | 1 | 0 | 0 |  |
| **HV9** | 2 | 2 | 1 | 0 | 0 | 1 |  |
| **HV10** | 2 | 2 | 2 | 0 | 0 | 0 |  |
| **HV11** | 1 | 0 | 0 | 1 | 0 | 0 |  |
| **HV12** | 2 | 0 | 0 | 2 | 0 | 0 |  |
| **HV13** | 6 | 0 | 3 | 3 | 0 | 0 |  |

**Supplementary Table S3.** Cycling conditions used for the amplification of immunoglobulin heavy chain sequences. PCR products were obtained in two rounds of semi-nested PCR using a G-Storm^TM^ 4 thermal cycler.

| **Step** | **T^0^C** | **Duration** | **Number of cycles** |
| --- | --- | --- | --- |
| Initial denaturation | 98^0^C | 120 s | 1 |
| Denaturation | 98^0^C | 10 s | 36 |
| Annealing | 60^0^C | 15 s |
| Extension | 72^0^C | 15 s |
| FINAL EXTENSION | 72^0^C | 60 s |  |
| Store | 4^0^C | ∞ (indefinitely) | 1 |

**Supplementary Table S4.** Semi-nested PCR primer sequences used in Rounds 1 and 2 from the immunoglobulin heavy chain PCR amplification.

| **Primer name** | **Sequence 5`🡪3`** | **Round** |
| --- | --- | --- |
| VH1L | CCATGGACTGGACCTGGA | Round 1 |
| VH2L | CAGATGGACATACTTTGTTCCAC | Round 1 |
| VH3L | CCATGGAGTTTGGGCTGAGC | Round 1 |
| VH4L | CGATGAAACACCTGTGGTTCTT | Round 1 |
| VH5L | ATGGGGTCAACCGCCATCCT | Round 1 |
| VH6L | GATGTCTGTCTCCTTCCTCAT | Round 1 |
| VH1F | CAGGTGCAGCTGGTGCAGTCTG | Round 2 |
| VH2F | GTCTTGTCCCAGGTCAACTTAAGGGAGTCTT | Round 2 |
| VH3F | GAGGTGCAGCTGGTGGAGTCTG | Round 2 |
| VH4F | CAGGTGCAGCTGCAGGAGTCGG | Round 2 |
| VH5F | GAGGTGCAGCTGCTGCAGTCTG | Round 2 |
| VH6F | CTGTCACAGGTACAGCTGCAGCAGTCAG | Round 2 |
| IGREV | CCAACTCTCTTGTCCACCTTGG | Rounds 1&2 |
